# High expression of TMEM200A is associated with a poor prognosis and immune infiltration in gastric cancer

**DOI:** 10.3389/pore.2023.1610893

**Published:** 2023-01-19

**Authors:** Hongyang Deng, Tengfei Li, Fengxian Wei, Wei Han, Xiaodong Xu, Youcheng Zhang

**Affiliations:** Department of General Surgery, Hepatic-Biliary-Pancreatic Institute, Lanzhou University Second Hospital, Lanzhou, China

**Keywords:** bioinformatics, gastric cancer, prognostic, immune infiltration, TMEM200A

## Abstract

**Background:** Gastric cancer (GC) is one of the global malignant tumors with high incidence and poor prognosis. Exploring new GC molecular markers is important to improve GC prognosis. Transmembrane protein 200A (TMEM200A) is a member of the family of transmembrane proteins (TMEM). This study is the first to investigate the potential function of TMEM200A and its relationship with immune infiltration in GC.

**Methods:** The differential expression of TMEM200A was determined through the Cancer Genome Atlas (TCGA) and Gene Expression Omnibus (GEO) databases. The receiver operating characteristic (ROC) curve was drawn to assess the diagnostic value of TMEM200A for GC. The relationship between TMEM200A and the clinical characteristics of patients with GC was investigated using the Wilcoxon test and the Kruskal-Wallis test. The effect of TMEM200A on overall survival (OS) was identified using the Kaplan-Meier method, the Log-rank test, the univariate/multivariate Cox regression analysis, and the nomogram prediction model. The co-expressed genes and gene set enrichment analysis (GSEA) were used to explore the potential biological functions of TMEM200A. We used the Tumor Immune Estimation Resource (TIMER) database and the ssGSEA algorithm to estimate the relationship between TMEM200A and immune cell infiltration. Furthermore, we investigated the correlation of TMEM200A with immune checkpoint/immune cell surface markers using the TCGA-STAD data set. Finally, we identified prognosis-related methylation sites in TMEM200A using MethSurv.

**Results:** TMEM200A was highly expressed in GC tissues. TMEM200A had a good diagnostic value for GC. High expression of TMEM200A may shorten the OS of GC patients and may be an independent risk factor for OS in GC patients. TMEM200A participates in the construction of a predictive model with a good predictive effect on the survival rate of GC patients at 1, 3, and 5 years. Co-expressed genes and GSEA indicated that TMEM200A may be an adhesion molecule closely associated with tumor invasion and metastasis. In addition, TMEM200A may be significantly associated with immune cell infiltration and immune checkpoint expression. We also found that TMEM200A contains three methylation sites associated with a poor prognosis.

**Conclusion:** Upregulated TMEM200A may be a promising prognostic marker for GC and is closely associated with the tumor microenvironment (TME).

## Introduction

Gastric cancer (GC) is common cancer worldwide, with more than one million new cases each year (1, 2). It has the sixth highest number of new cancers diagnoses in the world by 2020 (2). Furthermore, patients with GC are often diagnosed at an advanced stage and therefore do not have a high 5-year survival rate, making it the third leading cause of cancer-related death after lung and liver cancer (2). GC is most prevalent in East Asia, North East Asia, and North Africa (2). Surgery and platinum chemotherapy combined with fluorouracil are the mainstays of GC treatment, but the median overall survival (OS) after chemotherapy in patients with unresectable or metastatic GC is only less than 1 year (3). Despite the benefit seen with trastuzumab in combination with chemotherapy targeting the HER2 gene over chemotherapy alone (4), HER2 gene overexpression occurs in only 10%–20% of GC patients (5). Therefore, the development of new GC biomarkers or specific therapeutic targets may improve the prognosis of patients with GC.

The transmembrane protein (TMEM) family genes encode a large class of proteins that span the lipid bilayer of the cell membrane. Due to their transmembrane properties, TMEM proteins play a role in signal transduction, ion transport, and cell adhesion (6). Although some studies (7) suggested that genes in the TMEM family are essential for tumor metastasis. Different families of TMEM gene play cancer promoting or cancer suppressing roles in different cancers (8–10). In general, current knowledge of the biological functions and mechanisms of action of TMEM genes is limited (11). A deeper understanding of the function of TMEM genes may be a promising direction for the development of new therapeutic targets for tumors.

Transmembrane protein 200A (TMEM200A) (Ensembl ID: ENSG00000164484) is also a member of the TMEM gene family, located on chromosome 6q23.1, with a total length of 77.536 Kb and comprising 8 exons. Recent studies have shown that TMEM200A can regulate adipose morphology by affecting adipogenesis (12). Tan et al. (13) found that the expression of TMEM200A in familial pancreatic cancer was lower than in sporadic pancreatic cancer. Furthermore, 6q23.1, where TMEM200A is located, was identified to be associated with cleft palate and cleft lip (14). Bioinformatics analysis based on the Cancer Genome Atlas (TCGA) and Gene Expression Omnibus (GEO) databases showed that (15) TMEM200A could be used to construct a risk score for the prognosis of acute myeloid leukemia (AML) and could effectively predict the OS of patients with AML. Zhang et al. (16) analyzed somatic mutations and RNA prognostic markers in GC and initially found that TMEM200A may have a prognostic value in GC. However, the role of TMEM200A in GC is still unknown.

This study compared the differential expression of TMEM200A between GC tissues and adjacent/normal gastric mucosal tissues based on TCGA and GEO databases. Subsequently, Kaplan-Meier method, univariate/multivariate Cox regression analysis confirmed the effect of TMEM200A on the prognosis of patients with GC and used the expression of TMEM200A and clinical characteristics of patients with GC to construct a nomogram model to predict the prognosis. In addition, co-expression of genes and GSEA explored the possible biological functions and signaling pathways involved in TMEM200A. In addition, we calculated the relationship between TMEM200A and 28 immune cell infiltrates using the ssGSEA algorithm. We also explored the correlation between TMEM200A expression and immune checkpoint expression to further understand the impact of TMEM200A on the tumor microenvironment (TME). Finally, we analyzed the effect of TMEM200A on the sensitivity of common chemotherapeutic drugs and DNA methylation sites.

## Materials and methods

### Expression analysis of TMEM200A in pan-cancer

The Tumor Immune Estimation Resource (TIMER) database (https://cistrome.shinyapps.io/timer/) is based on the TCGA database (https://portal.gdc.cancer.gov/) of 10,897 cancer patients from 32 types of cancer and can be used to calculate differences in gene expression in cancer and adjacent tissues, correlations between genes and levels of immune cell infiltration (17). The GEPIA database (http://gepia.cancer-pku.cn/detail.php) contains gene expression data for 9736 tumor tissues from the TCGA database and 8587 normal tissues from the GTEx database (18). We used these two databases to assess the differential expression of TMEM200A in different kinds of tumor.

### Expression analysis of TMEM200A in GC

We downloaded RNA sequencing (RNA-Seq) data in TPM format for GC samples and adjacent samples from the TCGA database. The combined RNA-Seq data from the TCGA GC and GTEx databases of normal gastric tissue were downloaded from the UCSC database (https://xenabrowser.net/datapages/) (19), and the TCGA-STAD data set relating the clinical profile and survival information. In addition, we acquired GEO database to obtain GSE54129 and GSE66229 (20) as expression validation datasets. The receiver operating characteristic (ROC) curve was drawn to evaluate the diagnostic ability of the TMEM200A to identify GC tissue.

### Association of TMEM200A expression with clinical characteristics and prognosis of patients with GC

Excluding samples with partially missing data, we used RNA-Seq data for TMEM200A from 350 patients with GC in the TCGA-STAD dataset and the corresponding clinical information to assess the prognostic impact of TMEM200A. The median expression value of TMEM200A was used to divide the GC samples into high and low expression groups. We used Kaplan-Meier curves to visualize the effect of TMEM200A on the OS of patients with GC and calculated the *p*-value using the Log-Rank test. To evaluate the independence of the prognostic impact of TMEM200A, we performed a univariate/multivariate Cox regression analysis of patient clinical information such as age, sex, and stage with TMEM200A expression. Furthermore, we evaluated the efficacy of TMEM200A in predicting the prognosis of patients with gastric cancer using the nomogram prediction model and assessed the effect of the model by the C-index and the calibration curve. GSE15459 (21) contains RNA-Seq and survival information from 192 GC patients as an external validation cohort.

### Analysis of TMEM200A co-expressed genes in GC

The LinkedOmics database (http://www.linkedomics.org/login.php/) collects clinical characteristics, RNA-Seq, methylation and other multi-omics data from 32 TCGA cancer cohorts and 10 CPTAC cancer cohorts (22). We used it to calculate the co-expressed genes of TMEM200A, using volcano plot to represent all co-expressed genes and heat map to represent the top 50 genes positively and negatively correlated with TMEM200A. The Pearson correlation test was used to calculate the correlation coefficient, and genes with *p* < 0.05 and correlation coefficients greater than or equal to 0.3 were selected for Gene Ontology (GO) and Kyoto Encyclopedia of Genes and Genomes (KEGG) enrichment analysis by using the clusterProfiler (23) package R.

### Gene set enrichment analysis

MSigDB (http://www.gsea-msigdb.org/gsea/index.jsp) collects various annotated gene sets, and gene set enrichment analysis (GSEA) is an algorithm to calculate the variability of these gene sets between two different phenotypes (24). We made the log(TPM+1) normalized TCGA-STAD dataset as input RNA-Seq data and used TMEM200A as the phenotypic label and permutation tests 1000 times and considered statistically significant at *p* < 0.05 and FDR <0.05. The reference gene sets were c5.go.bp.v2002.1.Hs.symbols.gmt, c5.go.cc.v2002.1.Hs.symbols.gmt, c5.go.mf.v2002.1.Hs.symbols.gmt, and c2.cp.kegg.v2022.1.Hs. symbols.gmt. All results were sorted by NES value from largest to smallest.

### Correlation analysis between TMEM200A and immune cell infiltration

The TIMER database was used to estimate the relationship between TMEM200A and the abundance of six immune cell infiltrates. Subsequently, we further calculated the relationship between TMEM200A and 28 subtypes of immune cell infiltration by the ssGSEA algorithm of the GSVA (25) R package, and the results were presented in box plots. Finally, we explored the correlation of TMEM200A expression with immune checkpoint and immune cell surface marker expression in GC tissues using the TCGA-STAD dataset with adjacent excluded samples, and the results were visualised by radar plots.

### Drug sensitivity

We calculated the semi-inhibitory concentrations (IC50) of chemotherapeutic agents for each sample in the TCGA-STAD dataset using the pRRophetic R package. The patients were divided into high and low TMEM200A expression groups by median TMEM200A expression values and the difference in IC50 of these chemotherapeutic agents between the two groups was analyzed by the Wilcoxon test.

### Methylation analysis of TMEM200A

DNA methylation is a process of epigenetic modification of genes in which methyl groups are mounted on DNA cytosines. Aberrant DNA methylation plays a key role in cancer development. MethSurv (https://biit.cs.ut.ee/methsurv/) (26) is a database for DNA methylation survival analysis based on 7,358 patients of 25 different cancers in TCGA. We used MethSurv to analyze the methylation sites of TMEM200A and the impact of these methylation sites on the survival of patients with GC.

### Statistical analysis

The R software (version 4.1.2) and its packages were applied to test and visualize. *p*-values less than 0.05 were considered statistically significant.

## Results

### TMEM200A is aberrantly expressed in a variety of cancers

**FIGURE 1:**
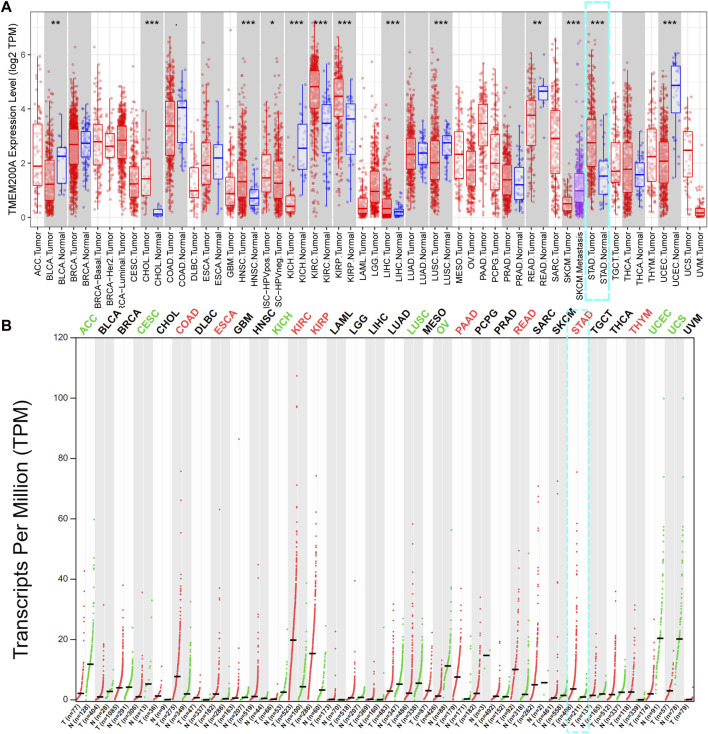
Pan-cancer analysis of TMEM200A expression levels. **(A)** Expression of TMEM200A in different kinds of tumor and adjacent tissues in the TIMER database. **(B)** Expression of TMEM200A in different kinds of tumor and normal tissues from the GEPIA database. *, *p* < 0.05; **, *p* < 0.01; ***, *p* < 0.001.

TIMER database analysis showed that TMEM200A was significantly more expressed in cholangiocarcinoma (CHOL), head and neck squamous carcinoma (HNSC), kidney renal clear cell carcinoma (KIRC), kidney renal papillary cell carcinoma (KIRP), liver hepatocellular carcinoma (LIHC), stomach adenocarcinoma (STAD) and less expressed in bladder urothelial carcinoma (BLCA), kidney chromophobe (KICH), lung squamous cell carcinoma (LUSC), rectum adenocarcinoma (READ), skin cutaneous melanoma (SKCM) and uterine corpus endometrial carcinoma (UCEC) compared to paraneoplastic tissues (Figure 1A). The results of GEPIA database analysis suggested that TMEM200A was highly expressed in colon adenocarcinoma (CODA), esophageal carcinoma (ESCA), KIRC, KIRP, pancreatic adenocarcinoma (PADD), READ, STAD and thymoma (THYM) and low in adrenocortical carcinoma (ACC), cervical squamous cell carcinoma and endocervical adenocarcinoma (CESC), KICH, LUSC, ovarian serous cystadenocarcinoma (OV), UCEC, uterine carcinosarcoma (UCS) compared to normal tissues (Figure 1B). Interestingly, we found that TMEM200A was significantly upregulated in GC tissues compared to both adjacent and normal tissues.

### TMEM200A is significantly highly expressed in GC and has diagnostic value

**FIGURE 2:**
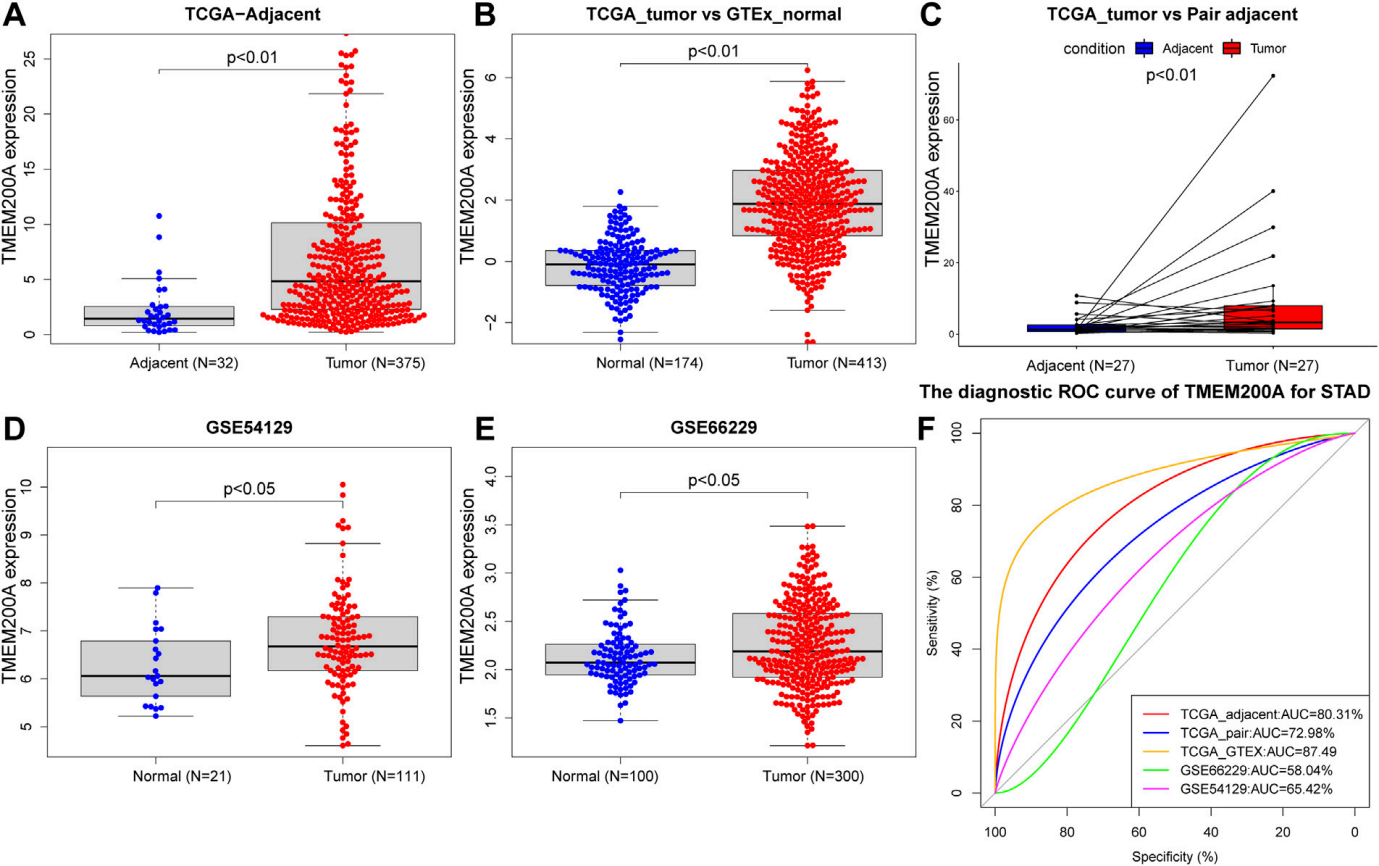
Expression level and diagnostic value of TMEM200A in GC. **(A)** TMEM200A expression levels in GC tissue (*n* = 375) and adjacent tissue (*n* = 32) in the TCGA-STAD data set. **(B)** Expression levels of TMEM200A in TCGA-STAD GC tissues (*n* = 413) and GTEx normal gastric tissues (*n* = 174). **(C)** TMEM200A expression levels in GC tissues (*n* = 27) and adjacent tissues (*n* = 27) of paired samples in the TCGA-STAD dataset. **(D)** Expression levels of TMEM200A in GC tissues (*n* = 111) and normal tissues (*n* = 21) of the GSE54129 dataset. **(E)** TMEM200A expression levels in GC tissues (*n* = 100) and normal tissues (*n* = 300) of the GSE66229 data set. **(F)** ROC curve of TMEM200A for identifying GC tissues.

After comparison, we further confirmed that the expression of TMEM200A in 375 GC samples was significantly higher than that in 32 adjacent tissues in the TCGA-STAD dataset (*p* < 0.01) (Figure 2A). Moreover, the expression of TMEM200A in 413 GC tissues was also significantly higher than that in 174 normal tissues (*p* < 0.01) (Figure 2B). Subsequently, the TCGA dataset of 27 paired GC and adjacent tissues (*p* < 0.01) (Figure 2C) and the GSE54129 (*p* < 0.05) (Figure 2D) and GEE66229 (*p* < 0.01) (Figure 2E) datasets also found that TMEM200A was significantly elevated in GC. ROC analysis revealed that TMEM200A had a strong value in the diagnosis of GC. The AUC of TMEM200A in TCGA-STAD, TCGA-STAD paired samples, TCGA-GTEx dataset, GSE54129 and GSE66229 dataset were 0.803, 0.730, 0.875, 0.580, and 0.654 (Figure 2F). These results indicated that TMEM200A was abnormally upregulated in GC.

### Correlation between TMEM200A and the clinical characteristics of GC patients

**FIGURE 3:**
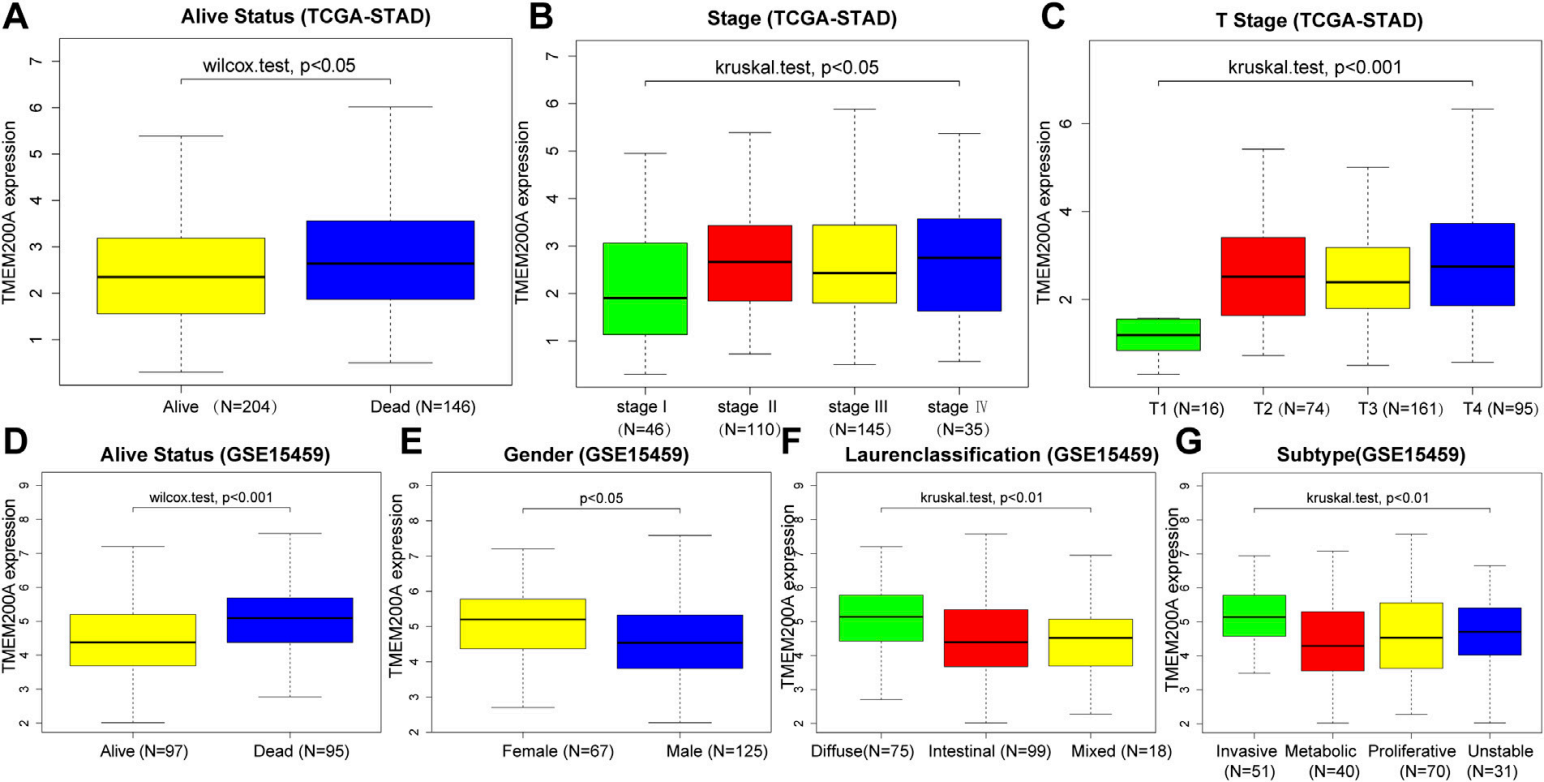
Correlation between the expression of TMEM200A and the clinical characteristics of patients with GC. TCGA-STAD cohort: **(A)** alive Status, **(B)** stage, **(C)** stage T. GSE15459 cohort: **(D)** alive Status, **(E)** gender, **(F)** Lauren-Classification, **(G)** Subtype.

Table 1 summarized the clinical characteristics of all GC patients in the TCGA-STAD and GSE15459 data sets. As shown in Figures 3A–G, highly expressed TMEM200A is distributed in dead patients (TCGA-STAD, alive vs. dead, *p* < 0.05) (GSE15459, alive vs. dead, *p* < 0.001) and female patients (GSE15459, female vs. male, *p* < 0.05). Furthermore, the expression of TMEM200A tends to increase with increasing clinical stage (TCGA-STAD, stage I vs. stage II vs. stage III vs. stage IV, *p* < 0.05) and T stage (TCGA-STAD, T1 vs. T2 vs. T3 vs. T4, *p* < 0.001). The distribution of TMEM200A expression varies between Lauren-classification and subtypes, with the highest expression in the diffuse and invasive types of GC. These results suggested that the high expression of TMEM200A appears to accelerate the progression of GC and contribute to its more aggressive nature.

**TABLE 1:** Clinicopathologic characteristics of GC patients in TCGA-STAD and GSE15459 datasets.

Characteristics	TCGA-STAD	GSE15459
	(N = 350)	(N = 192)
	N (%)	N (%)
Alive status		
Alive	204 (58.29)	97 (50.52)
Dead	146 (41.71)	95 (49.48)
Gender		
Female	124 (35.43)	67 (34.9)
Male	226 (64.57)	125 (65.1)
Age		
≥55	289 (83.29)	152 (79.17)
<55	58 (16.71)	40 (20.83)
Unkown	3	0
Stage		
I	46 (13.14)	31 (16.15)
II	110 (31.43)	29 (15.1)
III	145 (41.43)	72 (37.5)
IV	35 (10)	60 (31.25)
Unkown	14 (4)	0
Subtype		
Invasive	—	51 (26.56)
Metabolic	—	40 (20.83)
Proliferative	—	70 (36.46)
Unstable	—	31 (16.15)
Laurenclassification		
Diffuse	—	75 (39.06)
Intestinal	—	99 (51.56)
Mixed	—	18 (9.38)
Grade		
G1	9 (2.57)	—
G2	125 (35.71)	—
G3	207 (59.14)	—
Unkown	9 (2.57)	—
M stage		
M0	312 (89.14)	—
M1	23 (6.57)	—
Unkown	15 (4.29)	—
N stage		
N0	103 (29.43)	—
N1	93 (26.57)	—
N2	72 (20.57)	—
N3	71 (20.29)	—
Unkown	11 (3.14)	—
T stage		
T1	16 (4.57)	—
T2	74 (21.14)	—
T3	161 (46)	—
T4	95 (27.14)	—
Unkown	4 (1.14)	—

### Upregulated TMEM200A has GC prognostic value

**FIGURE 4:**
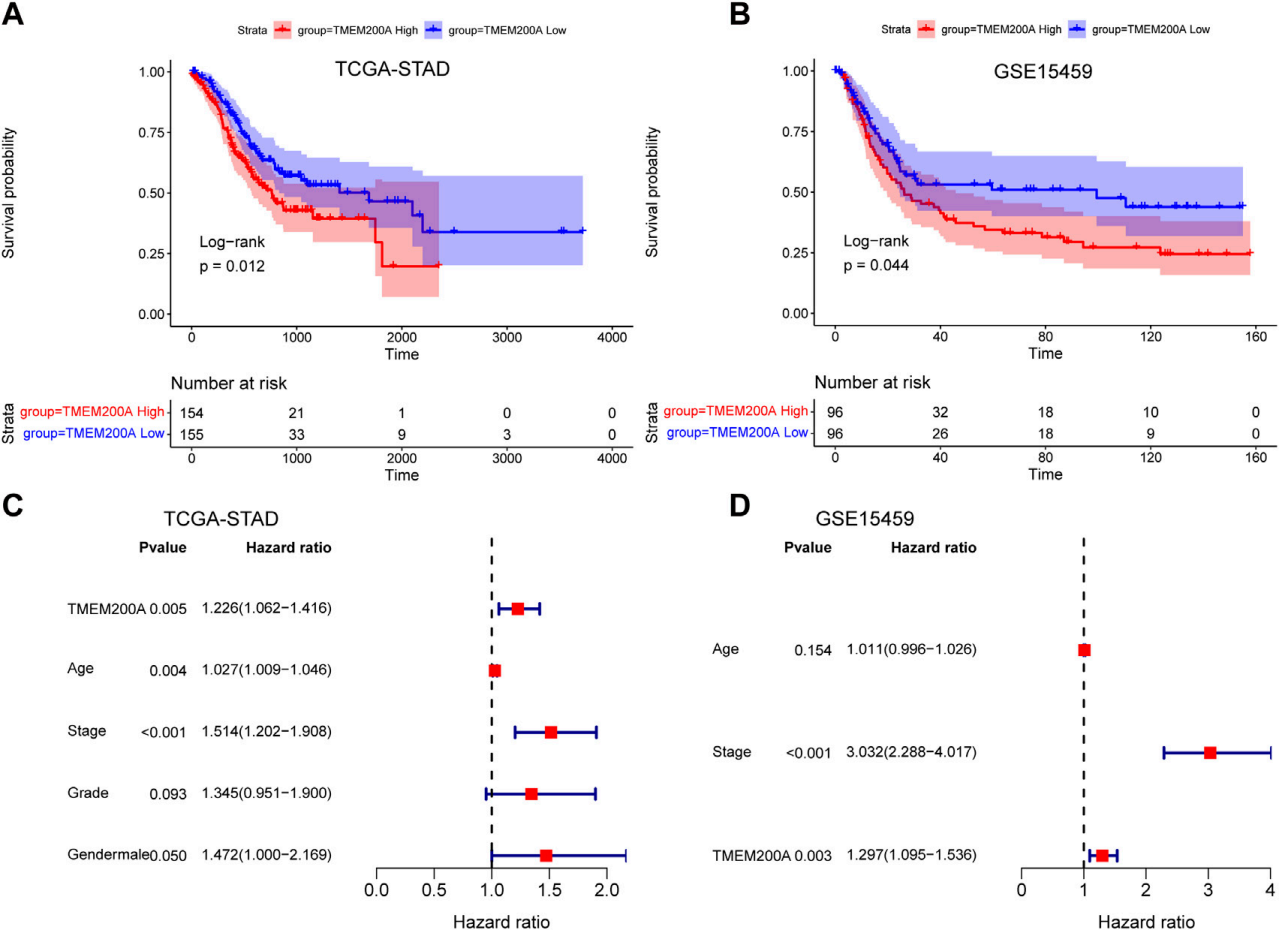
Analysis of the prognostic value of TMEM200A in GC. **(A)** Kaplan-Meier curves for OS of patients in the TCGA-STAD cohort. **(B)** Kaplan-Meier curves for OS of patients in the GSE15459 cohort. **(C)** Forest plot of the results of the TCGA-STAD cohort multifactorial Cox regression analysis. **(D)** Forest plot of the results of the multifactorial Cox regression analysis of the GSE15459 cohort.

**FIGURE 5:**
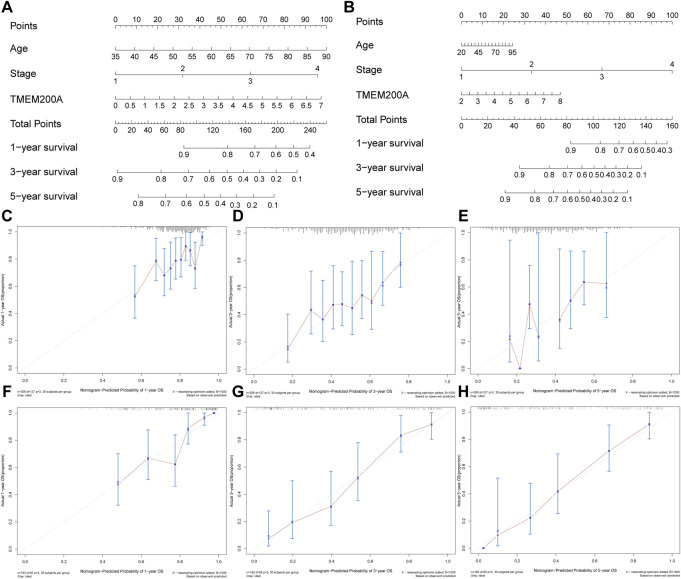
Nomogram plots of combining TMEM200A and clinical features evaluated survival in GC. **(A)** Nomogram for predicting OS in TCGA-STAD dataset. **(B)** Nomogram for predicting OS in the GSE15459 dataset. **(C–E)** The calibration curve of the nomogram for one, three, and 5 years of OS prediction in TCGA-STAD dataset. **(F–H)** The calibration curve of the nomogram for one, three, and 5 years of OS prediction in GSE15459 dataset.

In the TCGA-STAD cohort (*n* = 309), the calculation of the Kaplan-Meier method showed that patients with GC with high expression of TMEM200A had significantly lower OS than those with low expression of TMEM200A (*p* = 0.012) (Figure 4A). Univariable/multifactor Cox regression analysis illustrates that TMEM200A was an independent risk factor for OS in GC patients (HR = 1.226, 95%CI = 1.062–1.416, *p* = 0.005) (Figure 4C) (Table 2). Based on the results of multifactor Cox analysis, we selected factors with p less than 0.05 to construct a nomogram prediction model for predicting the survival rate of GC patients at 1, 3 and 5 years. The C-index of the model is 0.635 (95% CI = 0.608–0.662) (Figure 5A), indicating good predictive performance. The calibration curves show a good fit between the probability of predicting OS at 1, 3 and 5 years in GC patients and the actual probability of occurrence on the column graphs (Figures 5C–E).

**TABLE 2:** Results of a univariable/multivariate Cox regression analysis based on the TCGA-STAD dataset.

	Univariate cox regression		Multivariate cox regression	
Factors	HR (95%CI)	*p*-value	HR (95%CI)	*p*-value
TMEM200A	1.244 (1.082–1.431)	0.002	1.226 (1.062–1.416)	0.005
Age	1.021 (1.004–1.040)	0.017	1.027 (1.009–1.046)	0.004
Stage	1.474 (1.191–1.825)	<0.001	1.514 (1.202–1.908)	<0.001
Grade	1.377 (0.974–1.946)	0.070	1.345 (0.951–.900)	0.093
M Stage	1.675 (0.877–3.200)	0.118	—	—
N Stage	1.308 (1.118–1.530)	<0.001	—	—
T Stage	1.299 (1.040–1.622)	0.021	—	—
Gender	1.464 (0.998–2.147)	0.051	1.472 (0.999–2.169)	>0.05

Furthermore, we further validated the effect of TMEM200A on the survival of patients with GC by analyzing the GSE15459 dataset (*n* = 192). Similarly, GC patients with high TMEM200A expression have lower OS than those with low TMEM200A expression (*p* = 0.044) (Figure 4B). Meanwhile, TMEM200A is an independent risk factor for the prognosis of patients with GC (HR = 1.297, 95%CI = 1.095–1.536, *p* = 0.003) (Figure 4D) (Table 3). We also used TMEM200A in conjunction with age and stage to build the nomogram prediction model (Figure 5B), which also had excellent prediction performance (C-index = 0.766, 95%CI = 0.745–0.787) (Figures 5F–H). In conclusion, our study showed that upregulation of TMEM200A expression promoted GC progression and shortened the OS of GC patients.

**TABLE 3:** Results of a univariable/multivariate Cox regression analysis based on the GSE15459 dataset.

	Univariate cox regression		Multivariate cox regression	
Factors	HR (95%CI)	*p*-value	HR (95%CI)	*p*-value
TMEM200A	1.197 (1.010–1.420)	0.038	1.297 (1.095–1.536)	0.003
Age	0.999 (0.984–1.016)	0.969	1.011 (0.996–1.026)	0.154
Stage	2.790 (2.140–3.635)	<0.001	3.031 (2.288–4.017)	<0.001
Gender	1.402 (0.908–2.165)	0.127	—	—

### Co-expression gene analysis of TMEM200A

**FIGURE 6:**
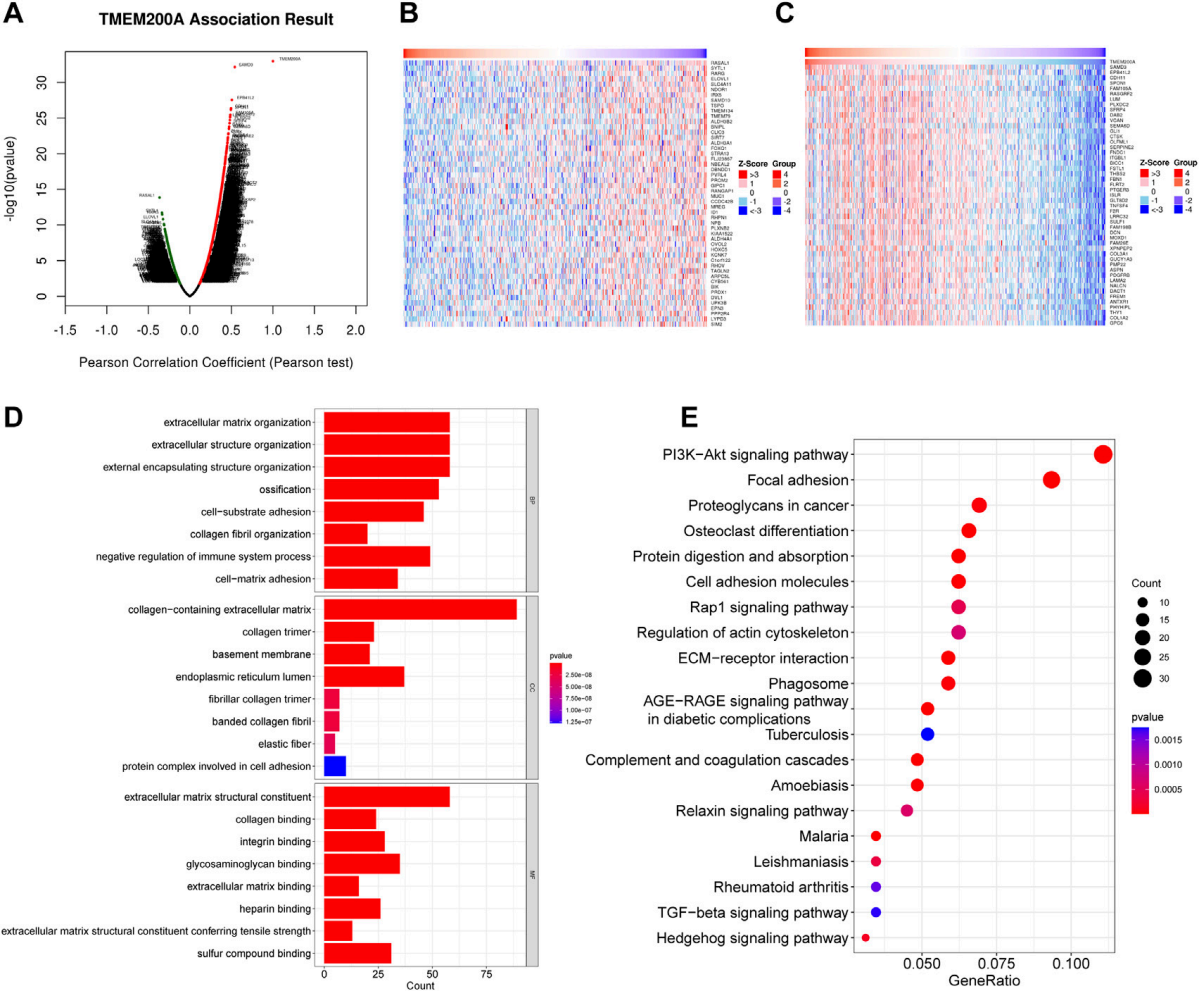
Co-expression of genes and functional enrichment of TMEM200A. **(A)** Volcano map of 8492 co-expressed genes of TMEM200A. **(B)** Heat map of the top 50 TMEM200A negatively related genes. **(C)** Heat map of the top 50 TMEM200A positively related genes. **(D)** GO enrichment analysis. **(E)** KEGG enrichment analysis.

LinkedOmics identified a total of 8492 TMEM200A related genes (Figure 6A) of which 3,074 were negatively related (Figure 6B) and 5,418 were positively related (Figure 6C). The total number of TMEM200A positively correlated genes with correlation coefficients greater than or equal to 0.3 was 705. In general, co-expressed genes have similar biological functions. The results of GO analysis suggested that the co-expressed genes of TMEM200A might be part of the cytoskeleton and were associated with ECM and adhesion (Figure 6D). KEGG pathway enrichment analysis revealed that co-expressed genes of TMEM200A could be involved in the P13K-Akt signaling pathway, focal adhesion, cell adhesion molecules, Rap1 signaling pathway, regulation of actin cytoskeleton, ECM-receptor interaction, phagosome, relaxin signaling pathway, TGF-beta signaling pathway, and Hedgehog signaling pathway (Figure 6E). These results suggested that TMEM200A may promote the progression of GC through these pathways.

#### GSEA

**FIGURE 7:**
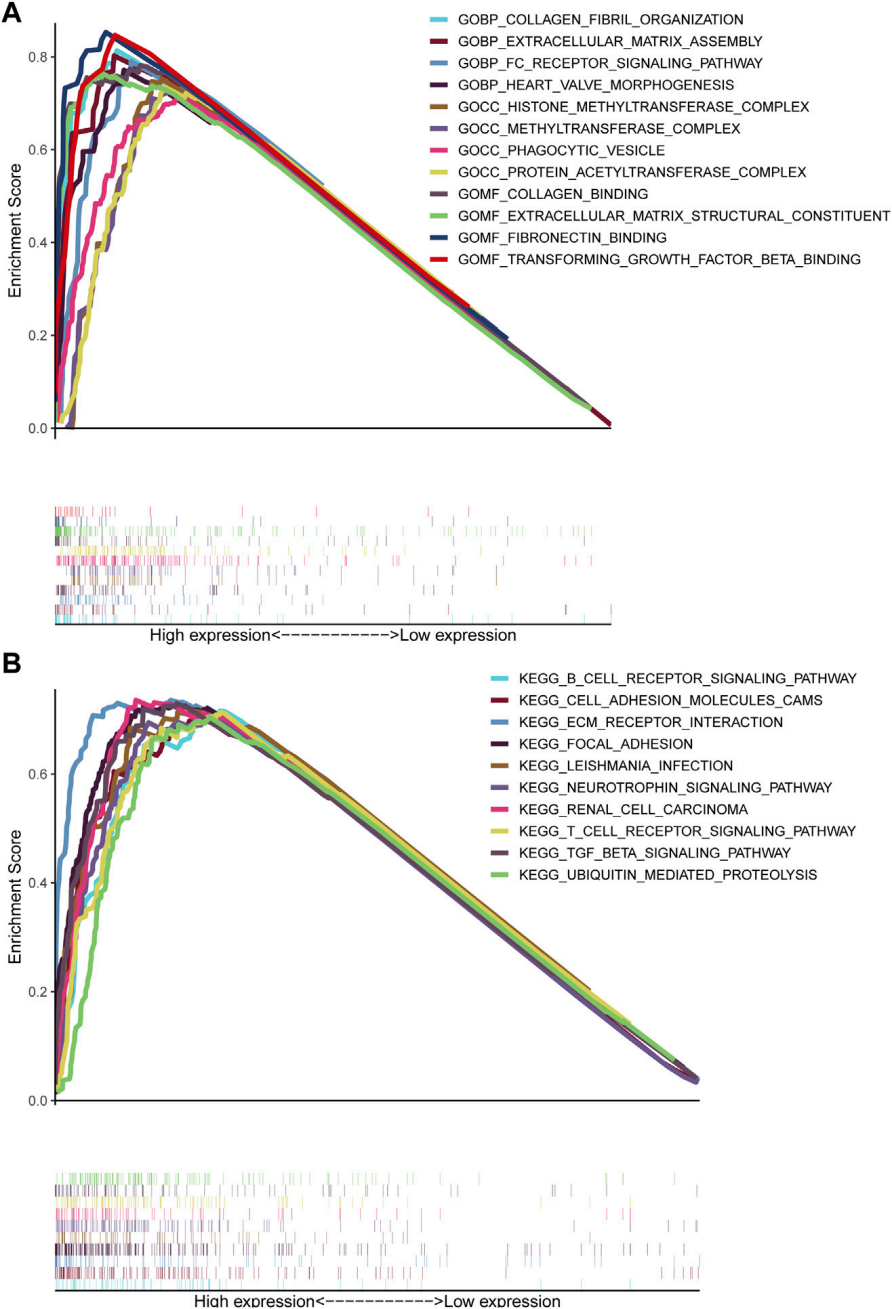
Results of the GSEA analysis of TMEM200A. **(A)** GSEA of GO. **(B)** GSEA of KEGG.

GSEA unraveled that extracellular matrix assembly, FC receptor signaling pathway, phagocytic vesicle, extracellular matrix structural constituent, fibronectin binding, transforming growth factor beta binding, B cell receptor signaling pathway, cell adhesion molecules CAMs, ECM receptor interaction, focal adhesion, T cell receptor signaling pathway, TGF-beta signaling pathway and ubiquitin-mediated proteolysis (Figures 7A, B), indicating that elevated TMEM200A could participate in the occurrence and progression of GC through these pathway. This is similar to the results of the co-expression gene enrichment analysis of TMEM200A, suggesting that TMEM200A may be an important adhesion molecule in extracellular information traffic and may be relevant to tumor immunity.

### TMEM200A is associated with immune cell infiltration in GC

**FIGURE 8:**
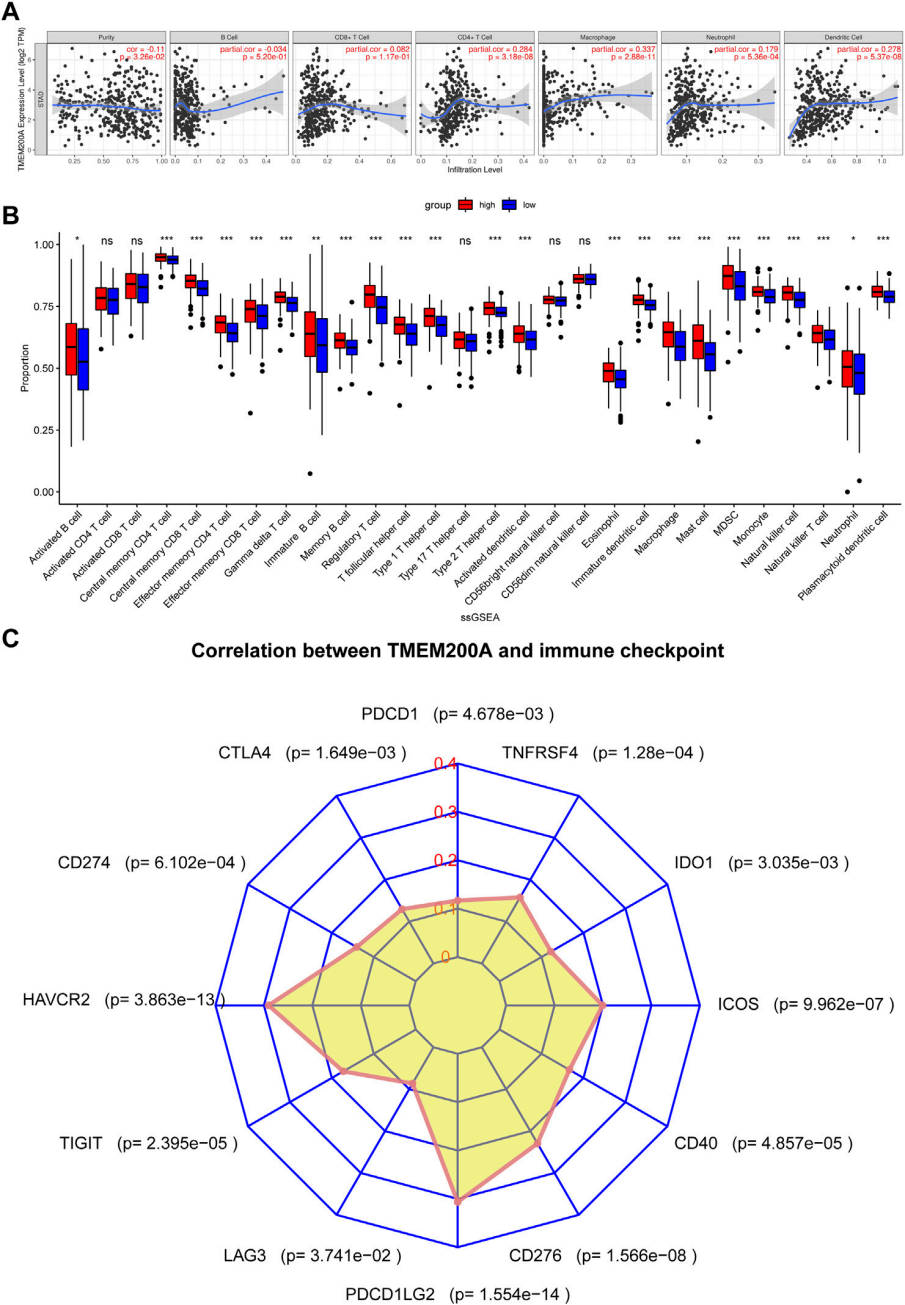
Results of TMEM200A and tumor immunological correlation analysis. **(A)** Correlation analysis of TMEM200A with the level of infiltration of six types of immune cells in the TIMER database. **(B)** Correlation analysis of TMEM200A with 28 immune cell subtypes calculated using the ssGSEA algorithm. **(C)** Correlation analysis of TMEM200A and immune checkpoint expression.

We found a positive correlation between TMEM200A and CD4^+^ T cells (*r* = 0.284, *p* = 3.18e−08), macrophages (*r* = 0.337, *p* = 2.88e−11), neutrophils (*r* = 0.179, *p* = 5.36e−04) and DC (*r* = 0.278, *p* = 5.37e−08) based on TIMER (Figure 8A). Furthermore, 23 of the 28 immune cell subtypes could be regulated by TMEM200A expression in GC tissues (Figure 8B). In addition, we found that TMEM200A is positively correlated with 17 common surface markers of the immune cell (Table 4). Interestingly, TMEM200A was positively correlated with the expression of 12 immune checkpoints in GC (Figure 8C), such as PDCD1, TIGIT, LAG3, CD40, etc. The above results suggested that TMEM200A probably modulated the tumor immune microenvironment (TIME) of GC, promoted the infiltration of immune cells and upregulates the expression of various immune checkpoints, which helped GC cells to immune escape and thus promoted the progression of GC.

**TABLE 4:** Results of correlation analysis of TMEM200A and immune cell surface markers.

Cell type	Marker	Cor	*p*-value
CD8^+^T cell	CD8A	0.174	**7.28E-04**
	CD8B	0.164	**1.45E-03**
CD4^+^T cell	CD4	0.37	**1.24E-13**
	CD40LG	0.276	**5.66E-08**
	CXCR4	0.287	**1.62E-08**
T cell (general)	CD2	0.254	**6.17E-07**
	CD3E	0.199	**1.09E-04**
	CD3D	0.184	**3.44E-04**
	CD6	0.25	**9.63E-07**
	SH2D1A	0.249	**1.09E-06**
	TRAT1	0.225	**1.07E-05**
	CD3G	0.24	**2.66E-06**
B cell	CD19	0.123	**1.76E-02**
	CD79A	0.136	**8.51E-03**
Monocyte	CD86	0.371	**1.12E-13**
	CSF1R	0.392	**3.19E-15**
TAM	CD68	0.057	2.71E-01
	CCL2	0.287	**1.51E-08**
	IL10	0.287	**1.47E-08**
M1 macrophage	IRF5	0.183	**3.79E-04**
	PTGS2	0.196	**1.37E-04**
M2 macrophage	CD163	0.385	**1.02E-14**
	VSIG4	0.347	**4.82E-12**
	MS4A4A	0.378	**3.57E-14**
Neutrophil	S100A12	0.028	5.91E-01
	CEACAM3	0.132	**1.06E-02**
	CCR7	0.208	**4.74E-05**
	FPR1	0.31	**9.07E-10**
	SIGLEC5	0.251	**8.12E-07**
	CSF3R	0.266	**1.78E-07**
	FCAR	0.198	**1.12E-04**
	FCGR3B	0.136	**8.19E-03**
Nature killer cell	KIR2DL1	0.078	1.31E-01
	KIR2DL3	0.118	**2.25E-02**
	KIR2DL4	−0.017	7.38E-01
	KIR3DL1	0.101	**4.97E-02**
	KIR3DL2	0.063	2.24E-01
	KIR3DL3	−0.083	1.11E-01
	XCL1	0.122	**1.85E-02**
	XCL2	0.139	**7.19E-03**
	NCR1	0.055	2.84E-01
DC	ITGAX	0.375	**5.38E-14**
	HLA-DPA1	0.18	**4.58E-04**
	HLA-DRA	0.2	**9.68E-05**
	HLA-DQB1	0.105	**4.23E-02**
	HLA-DPB1	0.15	**3.64E-03**
	CCL13	0.225	**1.09E-05**
	HSD11B1	0.407	**2.23E-16**
Th1	TBX21	0.167	**1.18E-03**
	TNF	0.063	2.20E-01
	STAT1	0.196	**1.29E-04**
	STAT4	0.269	**1.25E-07**
Th2	IL13	0.131	**1.11E-02**
	GATA3	0.126	**1.44E-02**
	STAT5A	0.325	**1.11E-10**
	STAT6	0.281	**3.14E-08**
Tfh	VSIG4	0.347	**4.82E-12**
Th17	STAT3	0.302	**2.32E-09**
Treg	TGFB1	0.324	**1.24E-10**
	STAT5B	0.455	**1.57E-20**
	CCR8	0.371	**1.17E-13**
	FOXP3	0.292	**8.44E-09**
T cell exhaustion	TIGIT	0.216	**2.40E-05**
	GZMB	0.113	**2.90E-02**
	TOX	0.302	**2.33E-09**
	HAVCR2	0.363	**3.86E-13**
	LAG3	0.108	**3.74E-02**
	CTLA4	0.162	**1.65E-03**
	PDCD1	0.146	**4.68E-03**

The meaning of the bold values is p<0.05.

### Drug sensitivity analysis

**FIGURE 9:**
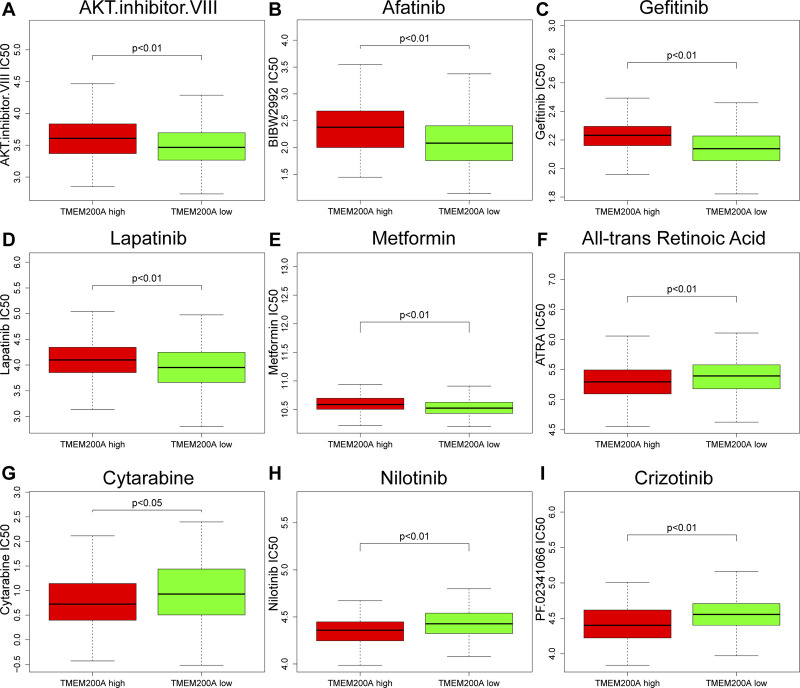
Correlation between the expression level of TMEM200A and the IC50 of chemotherapy drugs. **(A)** AKT.inhibitor.VIII, **(B)** Afatinib, **(C)** Gefitinib, **(D)** Lapatinib, **(E)** Metformin, **(F)** All-trans Retinoic Acid, **(G)** Cytarabine, **(H)** Nilotinib, and **(I)** Crizotinib.

After estimated the IC50 of 138 drugs in TCGA-STAD patients, we discovered that GC patients with high TMEM200A expression might gradually tolerate for AKT.inhibitor.VIII, Afatinib, Gefitinib, Lapatinib, and Metformin, while All-trans Retinoic Acid, Cytarabine, Nilotinib, and Crizotinib may have better therapeutic effects in these patients (Figure 9).

### DNA methylation sites of TMEM200A

**FIGURE 10:**
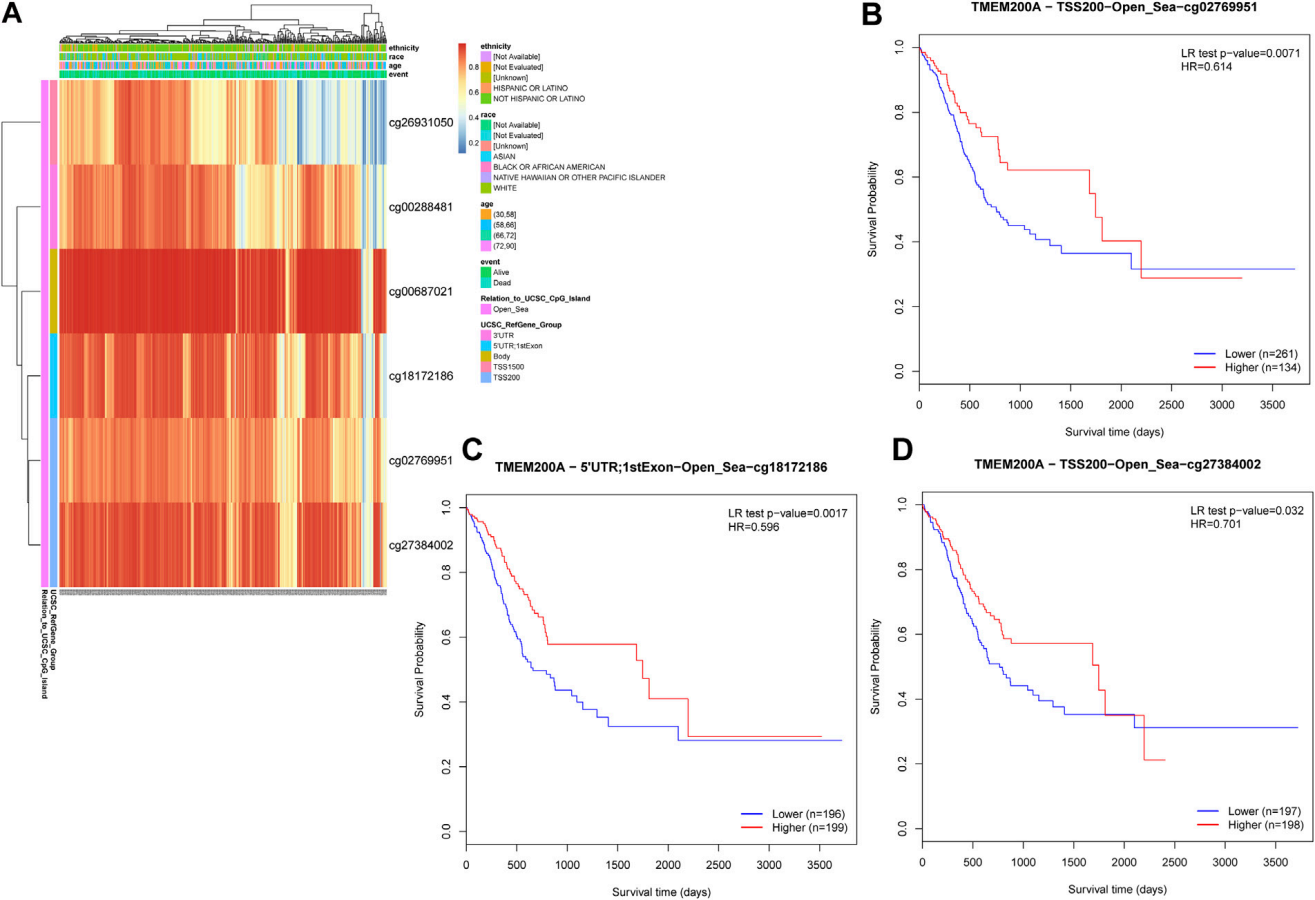
DNA methylation sites of TMEM200A. **(A)** Red to blue means that the methylation level goes from high to low. The different colored boxes represent ethnicity, race, age, event, relation to UCSC CpG Island, and UCSC RefGene Group. **(B)** The Kaplan-Meier curve of TMEM200A-TSS200-Open_Sea-cg02769951 in patients with GC. **(C)** The Kaplan-Meier curve of TMEM200A-5′UTR; 1stExon-Open_Sea-cg18172186 in patients with GC. **(D)** The Kaplan-Meier curve of TMEM200A-TSS200-Open_Sea-cg27384002 in patients with GC.

Six TMEM200A methylation sites were identified by using MethSury tool (Figure 10A), all of which had high methylation levels. Hypermethylation at three of these methylation sites was positively associated with a poor prognosis in patients with GC (Figures 10B–D). These results suggested that methylated of TMEM200A may be a potential method to inhibit the progression of GC.

## Discussion

Several members of the TMEM family gene have been shown to be associated with the progression of digestive cancers. For example, interference with TMEM45B expression inhibited the proliferation migration and invasive ability of GC cells (27). TMEM106C is highly expressed in LIHC and suppresses LIHC proliferation and metastasis after knockdown (28). High TMEM180 expression have been shown to predict low survival in COAD (29). Therefore, the intrinsic link between the members of the TMEM family gene and the tumors of digestive system deserves further exploration.

To the best of our knowledge, the expression and biological function of TMEM200A in GC and the relationship with TIME have not been reported. In this study, we found that TMEM200A was upregulated in GC tissues and shortened the OS of GC patients by calculating multiple data sets. Meanwhile, we also believe that the expression of TMEM200A increased with increasing staging. More importantly, univariable/multifactor cox regression analysis based on two independent data sets showed that TMEM200A was an independent risk factor for OS in patients with GC. Based on the results of multifactor Cox regression analysis, we constructed a nomogram prediction model using TMEM200A in combination with the clinical characteristics of GC patients, which had excellent predictive power for 1-year, 3-year and 5-year survival rates of GC patients. It is reasonable to assume that high expression TMEM200A promotes the progression of GC and is a valid prognostic indicator of GC. Therefore, it is necessary to further clarify the pathways through which it can exert its cancer-promoting effects.

Enrichment analysis of TMEM200A co-expressed genes revealed that TMEM200A could act as a cell membrane adhesion molecule possibly involved in the P13K-AKT pathway, focal adhesion, cell adhesion molecules, Rap1 signaling pathway, regulation of actin cytoskeleton, ECM-receptor interaction, phagosome, relaxin signaling pathway, TGF-beta signaling pathway, and Hedgehog signaling pathway. It has been shown (30) that the P13-AKT pathway can promote GC cells proliferation and invasion. Rap1, which has a high sequence similarity to the Ras oncoprotein, is an important Ras regulator and has been shown to be highly associated with cell adhesion and integrin function (31). Relaxin signaling promotes the proliferation, migration, invasion, and adhesion of prostate cancer cells (32) and is associated with ECM remodeling (33). The TGF-beta pathway is involved in the construction of an immunosuppressive microenvironment that assists in the immune escape of tumor cells (34). Organoid models of GC confirmed (35) that the Hedgehog signaling pathway mediates PD-L1 expression in GC cells *via* mTOR. Focal adhesion is part of the integrin adhesion complex and plays an important role in cell migration, cell cycle and cell proliferation (36). Similarly, cell migration is regulated by the cytoskeleton (37). In addition, it has been shown (38) that ECM is a key mediator of the metastatic spread of cancer cells. Overall, these findings demonstrated that TMEM200A may act as an adhesion molecule and be an accomplice in increased aggressiveness and metastasis of GC cells. These results were also corroborated by the GSEA results. Interestingly, we observed an additional enrichment of GSEA for the transforming growth factor beta binding, the B cell receptor signaling pathway, and the T cell receptor signaling pathway, suggesting that TMEM200A may be closely associated with the TIME.

The TIME, which includes tumor cells, immune cells and cytokines (39), has long been implicated in the progression, metastasis and recurrence of cancer (40). Our study found a positive correlation between TMEM200A and CD4^+^ T cell infiltration, macrophages, neutrophils, and DCs. CD4^+^ T cells are known to function through cytokines that help immune cells such as CD8^+^ T. However, CD4^+^CD25^+^ Treg cells, a subpopulation of CD4^+^ T cells, suppress effector T cells and thereby impair anti-tumor immunity (41, 42). Tumor-associated macrophages (TAM) are key regulators of GC progression (43). Especially, M2 TAMs promote GC angiogenesis and participate in the construction of TIME (43). Neutrophils are the main line of defense against exogenous agents, but their release of neutrophil extracellular traps (NETs) is one of the key regulatory players in promoting tumor recurrence and metastasis (44). In addition, we found that TMEM200A high expression samples were infiltrated by 23 more immune cell subtypes, which was also confirmed by correlation analysis of immune cell surface markers with TMEM200A. Collectively, these results suggested that TMEM200A is closely related to immune cells and likely induces immune cell infiltration that affects the prognosis of patients with GC.

Immune checkpoints are involved in creating an immunosuppressive microenvironment that promotes tumor immune escape. Our study revealed that TMEM200A is positively correlated with the expression of 12 common immune checkpoints, with HAVCR2, TIGIT, PDCD1LG2, CD276, and ICOS being the most significant. HAVCR2 (TIM-3) and TIGIT are co-inhibitory receptors expressed on T cells, Foxp3^+^ Treg cells, macrophages and DC cells that can cause T cell failure (45). PDCD1LG2 (PD-L2), the second ligand of PD-1, inhibits antitumor immunity by suppressing T-cell activation (46). It has been shown (47), that CD276 expression on the surface of tumor stem cells can promote their evasion of immune responses and epithelial mesenchymal transformation. T cells expressing ICOS can inhibit the proliferation of CD8^+^ T cells (48). These results demonstrated that although TMEM200A may induce immune cell infiltration in GC tissues. On the other hand, TMEM200A may inhibit antitumor immunity by up-regulating immune checkpoints.

Chemotherapy is a postoperative adjuvant treatment for patients undergoing surgery for GC and a dividend treatment for patients with advanced disease (5), but the ensuing resistance of tumor cells is also a cause of metastasis and recurrence (49). This study found that GC patients with high TMEM200A expression may be sensitive to All-trans Retinoic Acid, Cytarabine, Nilotinib, and Crizotinib, but may be resistant to AKT.inhibitor.VIII, Afatinib, Gefitinib, Lapatinib, and Metformin. These findings may help reduce drug resistance and improve the prognosis of GC patients. DNA methylation is considered to be one of the causes of tumorigenesis (50). As DNA methylation is reversible, regulation of DNA methylation has been suggested to be a promising direction for cancer therapy. Finally, we identified three TMEM200A methylation sites that were negatively associated with the prognosis of GC patients. Increasing the degree of methylation of these sites may provide new ideas for GC therapy.

## Conclusion

In conclusion, we found that TMEM200A was upregulated in GC and that high TMEM200A expression predicted poor prognosis. TMEM200A may play a role as an adhesion molecule regulating the GC immune microenvironment and promoting the invasive metastasis of GC cells. In addition, TMEM200A may upregulate immune checkpoints to help GC cells evade anti-tumor immune responses. Our study elucidated the potential role of TMEM200A in GC for the first time. We believed that TMEM200A deserves further study.

## Data Availability

Publicly available datasets were analyzed in this study. This data can be found here: All data generated or analysed during this study are available in the TCGA (https://portal.gdc.cancer.gov) and GEO (https://www.ncbi.nlm.nih.gov/geo/). The names of the repositories and accession number be found in the article/Materials and Methods, TCGA-STAD GSE54129, GSE66229, and GSE15459.

## Glossary

GC: gastric cancer
OS: overall survival
TMEM: transmembrane protein
TMEM200A: transmembrane protein 200A
TCGA: the cancer genome atlas
GEO: gene expression omnibus
ROC: receiver operating characteristic curve
TME: tumor microenvironment
TIMER: tumor immune estimation resource
RNA-Seq: RNA sequencing
CHOL: cholangiocarcinoma
HNSC: head and neck squamous carcinoma
KIRC: kidney renal clear cell carcinoma
KIRP: kidney renal papillary cell carcinoma
LIHC: liver hepatocellular carcinoma
STAD: stomach adenocarcinoma
BLCA: less expressed in bladder urothelial carcinoma
KICH: kidney chromophobe
LUSC: lung squamous cell carcinoma
READ: rectum adenocarcinoma
SKCM: skin cutaneous melanoma
UCEC: uterine corpus endometrial carcinoma
CODA: colon adenocarcinoma
ESCA: esophageal carcinoma
PADD: pancreatic adenocarcinoma
THYM: thymoma
ACC: adrenocortical carcinoma
CESC: cervical squamous cell carcinoma and endocervical adenocarcinoma
OV: ovarian serous cystadenocarcinoma
UCS: uterine carcinosarcoma
GO: gene ontology
KEGG: kyoto encyclopedia of genes and genomes
GSEA: gene set enrichment analysis
IC50: semi-inhibitory concentrations
TIME: tumor immune microenvironment
TAMs: tumor-associated macrophages
NETs: neutrophil extracellular traps
